# Knowledge on cervical cancer screening and vaccination among females at Oyibi Community

**DOI:** 10.1186/s12905-021-01296-3

**Published:** 2021-04-12

**Authors:** Evans Appiah Osei, Stella Appiah, Judith Elinam Gaogli, Ezekiel Oti-Boadi

**Affiliations:** 1 Department of Nursing, School of Nursing and Midwifery, Valley View University, P.O. Box DT 595, Oyibi, Ghana; 2 Valley View University, Oyibi, Ghana

**Keywords:** Knowledge, Cervical cancer, Screening, Vaccination, Females

## Abstract

**Background:**

Awareness about cervical cancer screening and vaccination in the developed countries are high as compared to the developing countries. Sixty to eighty percent (60–80%) of the women who develop cervical cancer in sub-Saharan Africa live in the rural areas with inadequate awareness of cervical cancer screening. However, cervical cancer knowledge remained a significant direct predictor of screening behaviors. The study therefore aim to explore the Knowledge on Cervical Cancer Screening and Vaccination among females at Oyibi Community.

**Methods:**

A qualitative exploratory design was employed to purposively recruit 35 participants who were made up of 7 members in a group forming 5 Focus Group discussions in all. Data was retrieved using a semi-structured interview guide.

**Results:**

The study revealed two main themes with 7 subthemes. The two main themes were cervical cancer screening and vaccination knowledge and cervical cancer vaccination effectiveness and cost. The subthemes were; knowledge on cervical cancer screening types, knowledge about cervical cancer screening and vaccination centers, knowledge about how cancer screening is performed, knowledge about cervical cancer vaccination, cervical cancer screening and vaccination sources of information, knowledge about the effectiveness of cervical cancer vaccination and awareness about cervical cancer screening cost and vaccination cost.

**Conclusion:**

Ghanaian women are increasingly becoming aware of cervical cancer, nevertheless low knowledge on screening and vaccination of cervical cancer, and effectiveness was detected with high awareness about the screening and vaccination centers. There is therefore the need for heightened sensitization regarding cervical cancer screening and vaccination in rural communities to help reduce misconceptions and increase patronage rate.

**Supplementary Information:**

The online version contains supplementary material available at 10.1186/s12905-021-01296-3.

## Introduction

Awareness about cervical cancer screening and vaccination in the developed countries are high as compared to the developing countries. For example, a study found that, majority of the respondents, 96.6% have good knowledge about cervical cancer screening, nevertheless the vast majority (98.9%) of the women have never been vaccinated against cervical cancer whiles 82.2% have never undergone cervical cancer screening [1]. It was revealed that, 91.2% of their participants knew that cervical cancer could be controlled and cited Pap-smear as a test to detect cervical cancer (80.9%) [2]. Similarly, some authors identified that, 30.4% of the respondents have not undergone cervical cancer screening because of fear of the screening procedure [3]. The major sources of information about cervical cancer screening and vaccination in the study were from friends and relatives as indicated by 42.8% of the respondents.

On the contrary, some studies have established that, the awareness level of cervical cancer screening and vaccination among Asians were low. For instance, in India, it was discovered that, more than half of their participants had poor knowledge regarding cervical cancer screening and vaccination [4]. Moreover, a study in India ascertained that, only 36% and 15% of their participants indicated they heard of HPV vaccine and cervical cancer screening respectively [5]. However, only 5% of these women had undergone a Pap smear and 4% of them felt they were at risk contracting HPV.

In Nigeria, a study revealed that, a little over half (51.8%) of the respondents had good knowledge about cervical cancer screening and vaccination with their sources mostly from school teachings [6]. However, some authors ascertained that knowledge of cervical cancer screening is likely not to translate into utilization of screening services in a sense that, their participants were found to have adequate knowledge on cervical cancer but had poor attitude towards the screening [7]. Some researchers in Hungary identified that some of their participants had poor knowledge regarding CCS and Vaccination [8]. In addition, more than half of women in one study gave incorrect answers for the benefits of the Pap smear such as cleaning of the womb, treatment of sexually transmitted infection and infertility [9].

In sub-Saharan Africa, the cervical cancer screening programs have a poor coverage [10]. For instance, a study stated that cervical cancer screening coverage in sub-Saharan Africa was 2 to 20% in the urban areas and 0.4 to 14% in the rural areas [11]. Sixty to eighty percent (60–80%) of the women who develop cervical cancer in sub-Saharan Africa live in the rural areas with inadequate awareness on cervical cancer screening and no opportunity of taking part in a cervical screening [12]. Also, cervical cancer screening programs has been found to have a better coverage among those of higher socio-economic class which still put the people of sub-Saharan Africa among those that would have poor coverage of the screening programs and vaccination utilization.

Similarly, awareness and knowledge of young women about cervical cancer and its related conditions and their willingness to screen are very important to the successful prevention against HPV and cervical cancer [13]. In Ghana, approximately 70% of the patients diagnosed to have the cancer are seen when their tumors have advanced to late stages [14]. Also most infections with HPV which leads to cervical cancer are asymptomatic and does not give early warning signs and symptoms for victims to seek for treatment. This, therefore makes widespread awareness and screening an important approach to preventing cervical cancer [13].

In 2012, it was discovered that among cancers affecting women in Ghana, cervical cancer is the commonest with an estimated 3,052 new cases and 1,556 deaths recorded [15, 16]. The Kumasi Cancer Registry in Ghana revealed that among females, cervical cancer (29.4%) was second most commonly diagnosed cancer after breast cancer (33.9%) [17]. According to some authors, about 8.6 million women 15 years and above are susceptible to Cervical cancer [18]. Annually, an estimated number of over 3000 cases of cervical cancer has been recorded with more than half thus over 2000 mortalities out of this number reported [19]. Cervical cancer screening utilization among women in Ghana have been found to be low [20, 21].

Early screening and vaccination has been suggested as an effective way of helping to reduce cervical cancer incidence [22–24]. Pap Smear and VIA methods are the commonly used and approved methods in Ghana [25]. Some researchers cited that nurses in Ghana have good knowledge regarding CCS and hence recommended the need for them to educate Ghanaian women in the Rural communities on it [26]. Aside the screening, vaccination against cervical cancer screening was approved in 2004 by Ministry of health to help reduce the burden of cervical cancer in Ghana [27]. Even though some barriers have been identified to prevent soem Ghanaian women from partaking in vaccination against HPV, some authors have discovered high willingness to vaccinate among Ghanaian women [27].

Nevertheless, in Ghana, a study among female students discovered that even though the participants had a fair perception of cervical cancer, they had a poor cervical cancer screening behavior [28]. The study further found that specific knowledge on cervical cancer and its risk factors as well as regular screening behavior is paramount to the prevention of cervical cancer. It Another study revealed that only perceived seriousness significantly mediated the relationship between cervical cancer knowledge and screening behaviors [29]. However, cervical cancer knowledge remained a significant direct predictor of screening behaviors. The study therefore aim to explore the knowledge on cervical cancer screening and vaccination among females at Oyibi Community.

## Methodology

A research design refers to the overall strategy that you choose to integrate the different components of the study in a coherent and logical way [30]. In this study, a qualitative exploratory design was employed. Qualitative research is a form of inquiry that analyzes information conveyed through language and behavior in natural settings [31]. It was used to capture expressive information that is not conveyed in quantitative data about beliefs, values, feelings and motivations that underlie behaviors. A focus group discussion was used in collecting data from participants since the researcher was interested in exploring the opinions of the women on cervical cancer screening and vaccination. It helped the researcher to retrieve varied responses about cervical cancer screening and vaccination.

A focused group discussion is a discussion which is carefully planned in which a purposefully set of participants are gathered to obtain perceptions on defined areas of interest in a permissive, non-threatening environment led through an open discussion by a skilled moderator [32]. The target population of this study included women living in the Oyibi community. The criteria for recruiting participants included women who were 18 years since women of such age range were at risk of cervical cancer, residing in Oyibi community, could express themselves in Twi and English and were willing to participate in the study.

A Purposive sampling was used for this study because all participants had the appropriate features for the study and they also provided information necessary for the study to ensure credibility. Sample size was based on data saturation. Each focus group discussion held was made up of 7 members. Interviews were conducted from one group to the other till no new responses were retrieved. Ethical clearance was obtained from the Dodowa Health Research Center Institutional Review Board (DHRC- IRB 31/03/20) which is one of the approved review boards in the country under Ghana Health Service and Ministry of Health that grants ethical clearance for various studies to be conducted throughout the country before the data collection. An approval letter from the ethical board to the assembly man and elders of the Oyibi community to seek their approval to gain entry into the community. Participants were selected based on the inclusive criteria. During the period of data collection, the researcher approached the females in various locations and gatherings such as churches, weddings, market places and houses within the community and invited them to participate in the study. The contacts of the participants were taken and interview days and venue were scheduled to suit participant’s availability. All the key ethical principles of informed consent, voluntary participation, anonymity and confidentiality were adhered to during the data collection process.

They were divided into a focus group of 7 members in each group and five focus group discussions were held, as the researchers served as a moderator for the interview given a sample size of 35 in all. The interviews were recorded with an audio tape. Notes were also be taken during data collection. The participants were asked to use initials to identify themselves to promote confidentiality. The interviews were conducted in English, Twi or Ewe depending on participant’s choice. Focus group interviews lasted for 45-60 minutes. Data was collected over a period of one and half months. The researchers thanked the participants for participating.

A semi-structured interview guide (Appendix A) attached as a Additional file 1 designed by the researchers was used to collect data from the focused group members. The interview guide consisted of open-ended questions with probes for further clarifications.

The recorded interviews were transcribed and saved on a personal laptop and it was secured with a password known only by the researcher. This was to ensure the safety of the interviews in case the laptop is stolen or breaks down

### Statistical analysis

Data were analyzed using content analysis as described as a descriptive presentation of qualitative data used to identify common patterns with the responds of participants and analyze them critically to achieve research aims and objectives [33]. The analysis was done after each discussion and continued throughout the data gathering process. Data collection and data analysis were done concurrently to help shape the data collection. Immediately after the end of each focus group discussion, the researcher played and listened to each discussion many times to familiarize herself with the data. The researcher then transcribed each interview verbatim. Each transcript was read and re-read for purposes of familiarization. The researcher then did color coding based on the meaning of the data using a same color for information having same meaning. Themes were written down and grouped based on patterns or relationships amongst them. A hierarchy of themes and sub-themes were created. Using a computer, similar themes were copied and placed in one file in line with the objectives of the study.

Rigor is emphasized in qualitative method because it generally determines the validity of the research done and reliability of the data generated, the extent to which results are represented and the subjectivity of research [34]. Credibility, transferability, dependability and confirmability have been identified as the major criteria for establishing trustworthiness in qualitative research [35].

## Results

### Socio-demographic characteristics of the participants

Thirty five participants constituting five (5) focus group discussions (FGD) were interacted with to obtain necessary data for the study. Each FGD consisted of seven (7) participants. The group consisted of only women from Oyibi Community within the Kpone-Katamanso District in the Greater Accra Region of Ghana.

The findings of the study revealed that majority of the participants (68.6%) were singles whilst few of them constituting 11 (31.4%) were married for 5 years and above. Concerning the age of participants, the results revealed that majority of the participants were within the age of 19 to 29 years with few above 40 years. Thus, the youngest participant was 19 years and the oldest participant was 60 years. The study again revealed that majority,that is, 15 participants of the respondents had secondary education (57.1%) and tertiary education 13 (37.1%) with 7 making the least participants having basic education background(20%). Majority of the participants 33 (94.3%) were Christians whilst a few of them (5.7%) were Muslims with diverse cultural backgrounds from the Volta region, Ashanti region, Eastern region, Greater Accra region, Northern region, and West region. Regarding their occupational status, majority of the participants were students(28.6%) government workers making up 25.7% of the employed participants interviewed and just a few(8.6%) were unemployed. Less than half of the participants(42.9) were nulliparous, with only one gravida 1 para 1 and the rest were multiparous; para 2, para 3 and para 5.

Two themes emerged from this study which were; Cervical cancer screening and vaccination knowledge and Cervical cancer vaccination effectiveness and cost.

### Cervical cancer screening and vaccination knowledge

From the analysis of data by the researcher, 5 sub-themes emerged under knowledge on cervical cancer vaccination and screening; Cervical cancer screening and vaccination knowledge and Cervical cancer vaccination effectiveness and cost knowledge on cervical cancer screening types, knowledge about cervical cancer screening and vaccination centers, knowledge about how cancer screening is done, knowledge about cervical cancer vaccination, cervical cancer screening and vaccination sources of information.

### Knowledge about cervical cancer screening types

This study revealed that the study participants had little knowledge with regards to cervical cancer screening and the types of the screening as reported in the quotes below.

I have not heard anything on cervical cancer screening types. Yeah I have a child but during my visits to the hospital when I was pregnant, they never said anything about the cervical cancer screening. Mostly the only thing they talked at the antenatal is how to examine the breast for breast cancer, that one dear they say it a lot but not this screening types you are talking about right now. (**P19)**

No please I have no ideas about what you are asking of. I don’t know of any effective and efficient screening that is done. The only thing I know of is the blood they take doing antenatal services. (**P35)**

For where I am, I don’t know much about other screening that is done; it is only pap-smear that I heard of but that one too I don’t know much about it and I have not done it myself, my friends told me about it. (**P9)**

Nevertheless few participants who happened to be health professionals demonstrated adequate knowledge on cervical cancer screening and types;

I am a nurse, so I have knowledge about cervical cancer screening types which is used to detect cervical cancer in women. The common types of screening we do in the hospital are the vaginal swap and the pap smearto investigate if there are papilloma virus at the cervix. These type of screening is better than the rest because you will get the screening results fast. The pap- smear is the most effective. I am a nurse and that what we mostly use to test people who visit our facility and I can tell you it is very effective so far. **(P4)**

I remember my friend who is a nurse said pap-smear is good to detect if you have the disease or not. My friend told me that it is not painful when screening for cervical cancer. (**P22)**

### Knowledge about cervical cancer screening and vaccination centers

The study reported that knowing where cervical cancer screening services is done is key, since it can positively impact the willingness to patronize the screening services. Majority of the participants were of the view that such services could be done in some government and private hospitals in the country.

Some private hospitals like our place Health Net offer those services, I think Nyaaho does it too and for the public hospitals, Korle bu, Ridge, and others also should be doing this screening since they are big hospitals. **(P8)**

It is said that government hospitals offer those services like Ridge and Korlebu. However, they don’t screen you for free. It is not part of the free vaccination so they said it goes for a price and it’s important to take anyways. **(P30)**

I am sure it will be done at the various hospitals but I don’t know which one does the screening and the administration of the vaccine. **(P13)**

However, few of the participants were totally ignorant about the various centers that offers cervical cancer screening.

My daughter as I was saying I don’t go to the hospital, I have never being to the hospital for all my life. So I do not know about any vaccine and the hospitals that does them. I have not even heard of the disease and the screening you were talking of (**P32)**

Please I do not know about any screening and vaccination center, however, there are many hospitals in the country but I do not know where in particular it is done.I don’t know about any hospital where those tests could be done. (**P29)**

### Knowledge about how cervical cancer screening is done

Some participants who happened to be nurse and few who have done it recounted that during the procedure a sample is taken from the cervix for analysis at the laboratory. The following quotes relates to the above results:

Cervical cancer screening is about investigations and screening of the reproductive system specifically the cervix of the woman to see if there is any disease that is if the human papilloma virus is there and it is done by taking a swab from the cervix by putting a speculum into the vagina in other to see the tip of the cervix. The nurse or doctor will educate you before a speculum is inserted to visualize the cervix. **(P4)**

Some participants had misconceptions that the screening cannot be done for virgins since it will cause them to loose their virginity and cause pain since their hymen is intact;

Moreover, the screening cannot be done for women who are virgins because it can devirginize a woman and cause pains. It would cause pain to them when the examiner tries to insert the instrument into the vagina since the hymen is still there and they will no more be virgins because there have been a penetration. **(P14)**

This screening cannot be done for virgins because they have not had any sexual intercourse with any man, so it will be of no use to screen for virgins since those that are at risk are women who have multiple sexual partners **(P22)**

Majority of the participants also reputed total ignorant about how the cervical cancer screening is done;

I don’t know how the screening is done like the step by step process because I have not done it before and I am not aware of it **(P3).**

I know about the condition and it is a cancer that affects women and we need to screen but I don’t know how the screening is done because I was not paying attention when the program was going on. I know that once a while you need to screen to know your status and if it is discovered that you have then they would treat you, so that you get better **(P20)**

### Knowledge about cervical cancer vaccination

Majority of the women were ignorant about the existence of the cervical cancer vaccine whereas some even doubted if a vaccine could prevent the occurrence of the disease. Some women also assume that cancer prevention vaccines were part of the childhood vaccinations.

I didn’t know there are vaccines available for preventing cervical cancer. I don’t have any idea about the vaccine but I think maybe it is part of the vaccination we were given when we were young like polio and the rest **(P24)**

I have heard of it but I don’t know if it is injection or poured into the mouth like the vitamin A vaccine given.**(P3)**

In addition, the findings brought to the light that a few of participants who knew about the vaccine believed that the cervical cancer vaccines fight against cervical cancer.

The vaccine is given to women that have gone through the test and have tested negative and treatment is given to those who tested negative. The vaccine is an injection given twice to prevent the disease. I was taught in school, and the clinic where I work does the screening and vaccination. **(P4)**

Yes, I know about the vaccine but I don’t remember the name though. I know that when the person is screened and the person is negative then the vaccine is given. And it protects you for life which is very good. The vaccine is not given to people who have tested positive even though I do not know why they do that. I think the good thing about this vaccine too is that when you are given it protects you for long. **(P8)**

### Cervical cancer screening and vaccination sources of information

Evidence gathered from the field data revealed that majority of the participants got to know about the cervical cancer screening and vaccination from various sources. The sources revealed in this study includes schools and relatives

As I said earlier that I am a Nurse and during my nursing training period we were thought about it and I remember one time some health workers came to our school during my nursing training to screen us but I didn’t do it because I wasn’t having money then **(P4)**

I got to know about the cervical cancer screening and vaccination from a relative during a family meeting. My cousin is a doctor and I remember during a family gathering he spoke to us about it. However, I watched it on television too and read more about it on the internet. He told us that he works at Ridge Hospital and his facility provides those services. **(P24)**

Few of the participants reported that they have not heard from any of the sources their colleagues were mentioning;

I do not know anything on cervical cancer screening and vaccination, not even during school time. I have not heard or seen it on television or Facebook or from friends. Yeah I have a child but during my visits to the hospital when I was pregnant, they never said anything about the cervical cancer disease or the screening and vaccination. Mostly the only thing they talked at the antenatal is how to examine the breast for breast cancer, that one dear they say it a lot but not this disease you are talking about right now. **(P19)**

### Cervical cancer vaccination effectiveness and cost awareness

The two subthemes that emerged are; knowledge about the effectiveness of cervical cancer vaccination and awareness about cervical cancer screening cost and vaccination cost.

### Knowledge about the effectiveness of cervical cancer vaccination

Exploration on the effectiveness of cervical cancer vaccination ascertained that majority were of the view that the vaccine were not effective and hence prevented them from patronizing it. The subsequent paragraphs give comprehensive findings on the subject matter:

Hmm, even though I heard about cervical cancer vaccination, I have not gone for the screening to receive vaccine for this disease, so I don’t know if it is effective. **(P1)**

In my view, I will say the vaccination will not be effective because they have been given vaccines since but people keep getting sick so I think it will not be different in the case of cervical cancer vaccines. **(P8)**

### Awareness about cervical cancer screening cost and vaccination cost

The results show that a few of the participants knew about the cervical cancer screening and vaccination cost while majority of the participants were ignorant about the cost involved in the screening and the vaccination. The findings revealed that the cervical cancer screening and vaccination cost depends on the facility visited. The following statements relates to the above findings:

Actually, the vaccine is an injection. The client takes two shots and one cost about 500 Ghana cedis but even with the cost, I think it depends one the hospital you visit. The private hospitals might take a bit more than that though. The screening is like 50 or 100 Ghana cedis but that one too, it is sometimes free when it’s the cervical cancer month celebration. (**P12**).

I think the vaccine is about 600 Ghana cedis at my work place so if you are taken it for two then its 1200 Ghana cedis. As for the screening, it’s free sometimes and sometimes too we charge 50 Ghana cedis. It is 50 Ghana cedis because we have this thing that we do example during breast cancer month we do it for free so same goes for the screening of cervical cancer **(P35)**

However, majority of the participants were ignorant about the cost of screening and vaccination;

I don’t know of the cost of the vaccine because I don’t even know about the vaccine. Moreover, the screening too, I don’t know the amount they take when you go to the hospital to screen. I also don’t know how the screening is done and how the vaccine is given…. yeah .No, no, no, I don’t know about the cost of the screening even though I have heard about it. For the vaccine, I don’t know the cost because I have not heard about it. I don’t know what is really done when you go to the hospital to get screened because my friends didn’t talk about it and I have not screened and vaccinated too. **(P23)**

## Discussion

### Cervical cancer screening and vaccination knowledge

#### Cervical cancer screening types

The findings of the present study revealed that, participants knowledge on cervical cancer help influence them to assess cervical cancer screening services. Majority of the participants in this present study reported to have little knowledge on the cervical cancer screening types. Among the few participants who demonstrated adequate knowledge on cervical cancer screening types, majority were health professionals (nurse and pharmacist) of which they mentioned vaginal swap, pap smear, scans as the key diagnostic test for cervical cancer. Among the three diagnostic test mentioned above, pap-smear was mostly known to participants in this present study. The findings in this present study is in consonance with a similar study conducted among nursing university students in Nigeria which revealed that, few of the participants about 23.1% identified pap-smear as a screening test which reflected in the poor utilization of cervical cancer screening among the students [36].

### Knowledge about cervical cancer screening and vaccination centers

Cervical cancer screening and vaccination centers was another sub-theme that emerged from the present study. The study reported that, knowing where cervical cancer screening and vaccination services is done is key, since it can positively impact the willingness to patronize the screening and vaccinations services but that was not the case in this present study. Majority of the participants in this present study were of the view that, such services could be done in some government hospitals like Korle-bu and Ridge hospital and private facilities like Nyaho hospitals in the country. However, among these participants, it was deduced that some were just guessing since they knew that screening are done in hospitals. Similarly, the findings in this present study is in similitude to a study conducted in India among females who reported cervical cancer screening and vaccination were conducted in hospitals since they are health associated [3]. However, they did not specify if it was government or private hospitals as shown in this present study.

### Knowledge about how cervical cancer screening is done

Findings of the current study also suggest that, few participants in this study who have undergone cervical cancer screening acknowledged that, they were educated on cervical cancer screening prior to the screening. Others narrated that, they received information on how cervical cancer screening is done from friends and on TV programs. These participants recounted that, during the procedure, a sample is taken from the cervix for analysis at the laboratory with the help of an instrument which was inserted into the vagina. A study done in Ghana revealed that, majority of the participants were well informed on were the screening is done in this country and added that, pap-smear was done with the use of a speculum then taken to the laboratory for further pathological investigations [26].

### Cervical cancer screening and vaccination sources of information

Participants who have done cervical cancer screening cited nurses and doctors as their source of information. This was followed by social media platforms such as whatsup, facebook, instagram and least was reproductive health education in girls club in secondary school and women fellowship meetings. In correlation to the present study findings, some resaerchers found in their study that, among young adults in Nigeria, the common sources of information on cervical cancer screening and vaccination were from television/radio, followed by print media, health campaigns, family or friends and health care providers [26]. This findings had media topping the list of sources of information because the population used for the study were young, hence, were social media inclined.

### Knowledge about cervical cancer vaccination

Concerning knowledge about cervical cancer vaccination, the study found that, majority of the women were ignorant about the existence of the cervical cancer vaccine, whereas, some few participant that knew about it were not sure whether the vaccine was give by mouth of injection.. Whereas, some women also assumed that, cancer prevention vaccines were part of the childhood vaccinations. In respect to the above findings, a study conducted in South Africa revealed that, cervical cancer cannot be prevented; this is even with vaccine and other participants did not know that, it is preventable by early screening and the use of the vaccine because they did not have adequate knowledge concerning the HPV vaccine [37]. In relation to this current study, it not surprising that, the participants that took part in the study did not believe that, cervical cancer could be prevent with the use of the HPV vaccine, hence, imparted negatively on HPV vaccine utilization. In contrast, majority of participants (69.5%) knew the HPV vaccine was available and can help prevent cervical cancer compared to 38.2% who stated condoms as a preventative measure instead of vaccine [38].

### Awareness about cervical cancer screening cost and vaccination cost

The awareness of the cost of the screening and vaccination is very key in enabling one to make a decision to or not to utilize those services. Concerning the cervical cancer screening cost and vaccination costs, the results show that, few of the participants knew about the cervical cancer screening and vaccination cost. Participants who knew about the cost recounted that, the vaccine is an injection and with each shot costing GH¢500 (86 USD) - GH600 (103 USD), hence, two shots of the HPV vaccine was equivalent to GH¢1000 (172 USD) - GH¢1200 (207 USD). The participants also reinstated that, the screening cost varies from GH¢50 - GH¢100. However, they also added that, the cost of the screening also differs from facility to facility and during cervical cancer month celebration which is usually in January, it is done for free. In correlation, a cross-sectional study done among female students in a Nigerian Tertiary Institution, revealed that, only 38 (13.0%) were aware of the HPV vaccine cost and perceived it as expensive [39]. In contrast to this study findings, a study done in Australia proved that, the cost of vaccine was effective and inexpensive and was assumed at a price of 115 USD (GH¢ 669) per vaccine dose making 230 USD (GH¢ 1338) for two shots. Participants saw the cost of HPV vaccine as effective in Australia because the government had funds in place to help sustain and motivate women financially to screen and vaccinate, hence, increasing the number of women who utilizes those services [40]. This implies that, the high cost of cervical cancer screening and vaccination in private hospitals in comparison to the government hospitals could be attributed to the fact that, government hospitals receive funds to help reduce the cost whiles private hospitals do not.

### Knowledge about the effectiveness of cervical cancer vaccination

Exploration on the effectiveness of cervical cancer vaccination ascertained that, majority were of the view that, they do not know whether the vaccine is effective because they have not gone for the screening to receive HPV vaccine for against cervical cancer. This implies that, effectiveness of HPV vaccine could better be determined by individuals who have undergone cervical cancer vaccination. Series of studies have also shown the effectiveness of cervical cancer vaccine in the quest of preventing cervical cancer occurrence. A study conducted among medical students of the University of Lagos, Nigeria, revealed that, most of the respondents supported vaccination of adolescent girls and were willing to recommend vaccination to colleagues/friends (82.1%) due to the fact that, they knew the effectiveness of the HPV vaccine against cervical cancer [6].

## Conclusion

The study revealed low knowledge on screening and vaccination of cervical cancer among women who participated in the study. Moreover, the perception about the effectiveness of cervical cancer vaccination was low, however, majority of the participants knew the various screening centers in the country where cervical cancer was done. There is therefore the need for heightened sensitization regarding cervical cancer screening and vaccination in rural communities to help reduce misconceptions and increase patronage rate.

## Supplementary Information

**Additional file 1:** Declaration.

## Data Availability

All data generated or analysed during this study are included in this published article [and its Additional information files].
