# Supplementary material for: Knowledge on cervical cancer screening and vaccination among females at Oyibi Community
Source: BMC Womens Health. 2021 Apr 12;21:148. doi: 10.1186/s12905-021-01296-3 (PMC8042702; doi:10.1186/s12905-021-01296-3)
Supplement: Supplementary file 1 — Additional file 1: Declaration. [file 12905_2021_1296_MOESM1_ESM.docx]

**APPENDIX A**

**SEMI STRUCTURED INTERVIEW GUIDE**

Dear respondent,

We are lecturers in the Department of Nursing and Midwifery, Valley View University, Oyibi. We are conducting a study on the topic **“Knowledge on Cervical Cancer Screening and Vaccination among females at Oyibi Community”**. This is for academic purpose, the information will be available to the researchers and findings will be published to inform knowledge on the subject matter and to add to literature. We therefore guarantee that your responses will be kept strictly confidential and anonymous without revealing any personal information.

Thank You.

**A. Biographic data of Respondents**

1. Tell me all about yourself

**Probes:**

- Age
- Marital status
- Years of marriage
- Educational background
- Occupation (what is the range of your salary)
- Religion
- Cultural background
- Parity

**B. Knowledge on Cervical Cancer Screening and Vaccination**

1. Tell me all you know about cervical cancer screening and vaccination?

**Probes:**

- What do you know about cervical cancer screening?
- What do you know about Cervical cancer Vaccination?
- Tell me about some sources of your information about cervical cancer screening and vaccination information you know?
- Where are the screening and vaccination for cervical cancer done in Ghana?
- Tell me about cervical cancer screening and vaccination cost?
- How is the screening and vaccination done?
- What is the appropriate screening interval for cervical cancer?
- How does formal knowledge influence a woman’s knowledge on cervical cancer?
- At what age should a woman begin cervical cancer screening?
- At what age should a woman receive vaccination for cervical cancer
- What do you know about cervical cancer vaccination?
- Share your views concerning the benefits of cervical cancer screening and vaccination?
